# Validation of the flemish CARES, a quality of life and needs assessment tool for cancer care

**DOI:** 10.1186/s12885-016-2728-9

**Published:** 2016-08-30

**Authors:** Bojoura Schouten, Johan Hellings, Elke Van Hoof, Patrick Vankrunkelsven, Paul Bulens, Frank Buntinx, Jeroen Mebis, Dominique Vandijck, Ward Schrooten

**Affiliations:** 1 Faculty of Medicine and Life Sciences, Hasselt University, Martelarenlaan 42, 3500 Hasselt, Belgium; 2 AZ Delta, Rode-Kruisstraat 20, 8800 Roeselare, Belgium; 3 Department of Experimental and Applied Psychology, Faculty of Psychological and Educational Sciences, Free University of Brussels, Pleinlaan 2, 1050 Elsene, Belgium; 4 Department of Public health and Primary Care, Faculty of Medicine, KU Leuven, Kapucijnenvoer 33, PB 7001, 3000 Leuven, Belgium; 5 Belgian Center for Evidence-Based Medicine (CEBAM), Kapucijnenvoer 33-blok J, 3000 Leuven, Belgium; 6 Jessaziekenhuis, Stadsomvaart 11, 3500 Hasselt, Belgium; 7 Faculty of Medicine and Health Sciences, Ghent University, De Pintelaan 185, 9000 Ghent, Belgium; 8 ICURO, Guimardstraat 1, 1040 Brussel, Belgium; 9 Centre Hospitalier de Cayenne, Rue des Flamboyants, B.P 6006, 97306 Cayenne Cedex, France

**Keywords:** Cancer, Psycho-oncology, Psychosocial, Quality of life, Needs assessment, Validation, CARES

## Abstract

**Background:**

The Cancer Rehabilitation Evaluation System (CARES) is a quality of life (QOL) and needs assessment instrument of US origin that was developed in the 90’s. Since November 2012 the copyright and user fee were abolished and the instrument became publicly available the present study aims to reinvestigate the psychometric properties of the CARES for the Flemish population in Belgium.

**Methods:**

The CARES was translated into Flemish following a translation-back translation process. A sample of 192 cancer patients completed the CARES, concurrent measures, and questions on socio-demographic and medical data. Participants were asked to complete the CARES a second time 1 week later, followed by some questions on their experiences with the instrument. Internal consistency, test-retest reliability, content validity, construct validity, concurrent validity and feasibility of the CARES were subsequently assessed.

**Results:**

The Flemish CARES version demonstrated excellent reliability with high internal consistency (range .87–.96) and test-retest ratings (range .70–.91) for all summary scales. Factor analysis replicated the original factor solution of five higher order factors with factor loadings of .325–.851. Correlations with other instruments ranging from |.43|–|.75| confirmed concurrent validity. Feasibility was indicated by the low number of missing items (mean 2.3; SD 5.0) and positive feedback of participants on the instrument.

**Conclusions:**

The Flemish CARES has strong psychometric properties and can as such be a valid tool to assess cancer patients’ QOL and needs in research, for example in international comparisons. The positive feedback of participants on the CARES support the usefulness of this tool for systematic assessment of cancer patients’ well-being and care needs in clinical practice.

**Trial registration:**

ClinicalTrials.gov: NCT02282696 (July 16, 2014).

**Electronic supplementary material:**

The online version of this article (doi:10.1186/s12885-016-2728-9) contains supplementary material, which is available to authorized users.

## Background

Cancer is a disease with a huge impact on patients and their relatives, going far beyond the physical aspects. Together with the rise of more successful therapeutic approaches and the increased life expectancy, the psychological and social aspects of care receive more attention as part of a holistic view of health care. Health care, and certainly cancer care, therefore requires a more integrated approach as a response to the fragmented delivery of health and social services [1]. Together with more integration, health is moving towards a more patient-centered approach. This is a process evolution as patient-centered care is an important dimension of quality of care [2]. Individualized, more integrated care plans and clinical care pathways are developed to improve outcomes for cancer patients, with an increasing emphasis on quality of life (QOL) [3].

To integrate the psychosocial approach into cancer care, the implementation of routine psychosocial screening and needs assessment is recommended by international cancer systems and in guidelines [4–9]. However, not all patients with a positive screen for distress or decreased QOL are interested in professional support [10]. In some cases programs involving systematic or routine screening for distress lead to a considerable number of unaccepted referrals [11, 12]. In contrast to QOL or distress screening, needs assessment not only focuses on identifying patients’ unresolved concerns and problems, but furthermore explores whether or not there is a desire extra help [13]. This not only gives guidance from the patients’ perspective for more integrated and holistic care plans, but also allows for the more effective and efficient use of resources. [10].

The Cancer Rehabilitation Evaluation System (CARES) is a self-administered QOL and needs assessment instrument that can be used for research or clinical purposes [14–20]. The instrument covers a broad range of topics relevant to the QOL disruption many cancer patients experience. The CARES consists of 139 items meant to reflect the multidimensional burden of cancer and its treatment can cause to patients and their relatives. The items can be scored broadly using the six summary scales medical interaction, physical, psychosocial, marital and sexual wellbeing and miscellaneous items; or in a more detailed manner grouped under 31 subscales. However, not all items apply to all patients and therefore patients can complete a minimum of 93 items or a maximum of 132 items. Patients can rate each item, formulated as problem statement, on a five-point scale, zero representing “not at all” (no problem) and four representing “very much” (severe problem). For every applicable problem statement patients are asked to answer the question “Do you want help?” by ticking the box ‘yes’ or ‘no’.

The psychometric robustness of the CARES and its’ earlier development versions called the Cancer Inventory of Problem Situations (CIPS) are well documented and positively evaluated [17, 18]. With high Crohnbachs alpha’s (α = 0.87–0.94) and high test-retest correlations (*r* = 0.84–0.95) for the summary scales and CARES total the instrument demonstrates excellent reliability. The validity of the CARES was also rigorously tested. Results from post-administration interviews supported the content validity of the instrument [18, 21]. An extensive evaluation of concurrent validity was conducted with the Symptom Checklist-90 (SCL-90) [22], Dyadic Adjustment Scale (DAS) [23], Karnofsky Performance status Scale (KPS) [24, 25] and a visual analogue scale [26] for QOL before and after cancer, resulting in moderate to high correlations. In two studies investigating the feasibility of the CARES for patients, the participants on average needed 18 to 20 min to complete the CARES. The majority of them thought the questionnaire reflected relevant day-to-day problems of cancer patients; they understood the instructions well and found questions easy to understand and not offensive [18]. Despite this good quality the widespread use of the CARES and it’s short form was limited by copyright and a user fee that the developers chose to impose. Since November 2012 this is no longer the case [27].

Due to the combination of feasibility for patients, psychometrical robustness and the wide representation of life domains that can be disrupted by a cancer diagnosis and the side effects associated with treatment, the CARES was chosen for further research on QOL and care needs in Belgium. However, time perspective, culture and language are important for the ecological dimension and validity of an instrument [28]. Careful translation and validation of an instrument are extremely important for the data to be valid [29, 30]. Consequently, a validation study on the CARES was conducted in the Flemish-speaking part of Belgium. The thorough validation-exercise is described in this article.

## Methods

The protocol of this study, including a priori hypotheses and criteria, is described in detail in a previous publication [31]. The procedures used the general principles of scale development according to classical test theory.

### Participants

There are no general criteria for the sample size in a validation study, but a sample size of at least 50–100 is generally recommended [32]. Sample sizes in the validation research of the original CARES varied for each psychometric quality from 22 to 1047 [18]. In this validation study of the CARES, the objective was set to include at least 150 participants.

A heterogeneous sample of cancer patients was recruited in several departments of four Flemish hospitals from March 2014 to February 2015. Non-palliative cancer patients aged between 25 and 60 years with a primary diagnosis of Stage I, II or III cancer [33], were included. The age restriction was chosen in the belief that these adult cancer patients have a psychosocial context which is clearly different from that of younger and older patients by means of significant relationships with children, partners, parents and the work context. There were no exclusion criteria with regards to sex, performance status or topology of the cancer. Patients were excluded from the sample if they lacked basic proficiency in Dutch, had cognitive problems or a history of major neurological disease.. Patients signed an informed consent form before participation.

### Questionnaires

Participants had to complete two questionnaire bundles, within an interval of 1 week.

Data collected with the *first questionnaire bundle* included socio-demographic characteristics, medical characteristics, the CARES and seven concurrent instruments to assess concurrent validity.

### Flemish CARES version

The Flemish CARES version was produced through a forward-backward translation process with two sworn translators and an expert group.

In the ongoing study missing response categories for items 18 and 80 in the CARES were noticed, causing structural (non-random) missing answers (55.7 % of the analyzed questionnaires). A second and corrected version was printed and replaced the first (44.3 % of the analyzed questionnaires). To avoid possible bias, items 18 and 80 were excluded from analysis.

*Karnofsky Performance status Scale* (*KPS*) [24, 25, 34]: The KPS is an 11-point scale to evaluate the physical and daily functioning of a patient, ranging from 0 (completely dependent, not able to care for oneself) to 100 (fully active, not dependent and capable of normal activity without limitations).

*Hospital Anxiety and Depression Scale* (*HADS*) [35, 36]: The HADS was developed to identify symptoms of anxiety and depression in medically ill patients. The questionnaire contains 14 items with four response categories, ranging from 0–3. Higher scores on the two subscales (each consisting of 7 items) indicate a higher level of anxiety or depression and the total score of the HADS (score-ranges from 0–42) can be used as a global measure of psychological distress [37].

*Social Support List*-*Interactions and Discrepancies* (*SSL*-*I and*-*D*) [38–40]: The SSL is a questionnaire with 75 items, 41 on experienced social interaction and 34 on experienced social discrepancies. In the first part of the questionnaire participants indicate how frequently certain social interactions occur on a 4-point Likert scale from 1 (‘seldom or never’) to 4 (‘very often’), with higher scores representing higher levels of social support. A second part of the SLL indicates the social discrepancies participants experience ranging from 1 (‘I would like it to happen more often’) to 4 (‘it happens too often’). Higher scores on the SSL-D indicate a greater lack of social support.

*Maudsley Marital Questionnaire* (*MMQ*) [41–43]: The MMQ contains three scales exploring Marital (10 items), Sexual (five items) and General Life (five items) adjustment. The items of the MMQ are scored on a 9-point Likert scale (ranging from 0 to 8). The wording of response categories differs for each item depending on the nature of the question.

*European Organisation of Research and Treatment for Cancer Quality of Life Questionnaire Core 30* (*EORTC*-*QLQ*-*C30*) [44]: The EORTC QLQ-C30 is a cancer-targeted quality of life instrument, incorporating five functional scales (physical, role, cognitive, emotional and social) and three symptom scales (fatigue, pain and nausea, and vomiting). Items are scored on a 4-point Likert scale from 1 (‘not at all’) to 4 (‘very much’). The last two items on global health and quality-of-life have an 8-point Likert scale, ranging from 1 (‘very poor’) to 7 (‘excellent’).

*Distress Thermometer* (*DT*) *together with a Problem List* (*PL*) [45–47]: Patients are asked to rate their overall distress on a visual analogue scale (presented as a thermometer) from 0 (‘no distress’) to 10 (‘extreme distress’). The DT is accompanied by a Problem List, which includes 35 items that address 5 life domains (practical, family/social, emotional, spiritual, and physical problems). Participants indicate if the stated problems apply to them. At the end of the survey participants are asked if they want to talk to a professional about their problems.

*Care Needs Questionnaire* [48]: The Care Needs Questionnaire was developed by Pauwels and Van Hoof to assess the care needs of cancer patients regarding specific themes during reintegration: physical functioning, psychological functioning, self and body image, sexuality, relationship with partner, relationship with others and work and social security related aspects. For each theme, participants are asked whether they wish to receive information or support, how they prefer to receive information and support, and to what extent this need already has been met. Each of the questions are answered on a 3-and 4-point Likert scale with different wording.

The *second questionnaire bundle*, filled in a week after the first one, contained the CARES and supplementary questions on patients’ experiences with the CARES in relation to the importance and breadth of issues assessed, length of time to complete, and format of survey administration.

### Study procedure

**Fig. 1:**
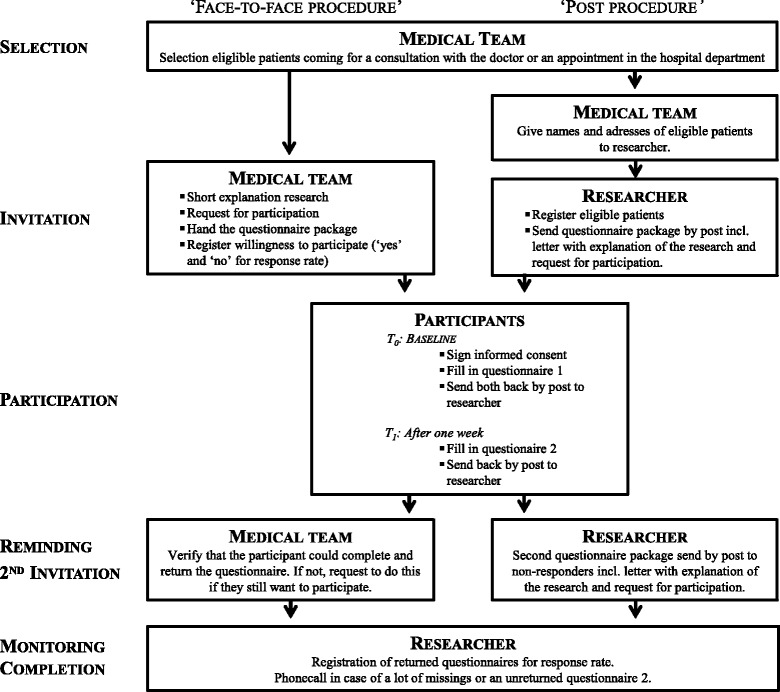
Study procedure

Eligible patients were selected by the medical team according to the inclusion and exclusion criteria [49]. On the basis of team organization and time availability, two alternative procedures to invite patients to participate in the study were used (Fig. 1).

In the ‘face-to-face procedure’, a member of the medical team explained the study briefly and invited the patient to participate. If the patient agreed, he/she immediately received a study package with the informed consent form, a ‘what to do’-scheme, the first questionnaire bundle and a stamped and addressed envelope to return the questionnaire.

In the ‘post procedure’, eligible patients got sent an identical study package by post, plus a letter explaining the study. One week later participants had to complete the second questionnaire bundle and send it back in another stamped and addressed envelope provided.

If the questionnaire was not sent back, the participants recruited via the face-to-face procedures were contacted by a team member. Participants invited through the post procedure were sent a reminder and second questionnaire package after 1 month. The researcher contacted participants by phone or by e-mail when returned questionnaires had a large number of missing responses or if the second questionnaire was not received in the expected timeframe. Since ethical standards limit the number of participant contacts, there was a maximum of two attempts to contact a participant.

### Data analysis

The Statistical Package for Social Sciences (SPSS; Chicago, IL) version 22.0 was used for statistical analyses of the data.

Descriptive statistics were used to analyze socio-demographic and medical data, as well as the data gathered with the supplementary questions from the second questionnaire bundle.

The *reliability* of the CARES was explored by the internal consistency of summary scales, with the aim to find a Cohen’s Alpha of at least .70 [50, 51]. Test-retest reliability was investigated by computing Spearman’s rho correlations between the summary scale scores and total-CARES scores of the first and second CARES administration, requiring a correlation ≥ .70 [50, 52].

Principal component analysis (PCA) and inter correlations of summary scales were computed to evaluate *construct validity*. Due to the complexity of the CARES, number of items and items only applicable for a subgroup of the sample, one general factor analysis on all the individual items was not possible in this small sample. PCA with varimax rotation was used in two subsequent analyses to assess the underlying factor pattern of the Flemish CARES. A first PCA was carried out on the individual items of the five summary scales to explore the CARES subscales. A higher order (second-order) factor analysis on the 26 subscales was conducted to explore the five summary scales. As in previous CARES-research items and subscales with a factor loading higher than .30 were seen as loading on a factor [17, 18].

Spearman’s rho correlations were computed to evaluate *concurrent validity* of the CARES global score and the summary scales with the seven concurrent instruments. Correlations were judged low, moderate and high, when their absolute values were respectively < .30, from .30–.50 and ≥ .50 [53].

## Results

### Sample characteristics

With 197 of the 325 invited patients returning completed questionnaires the response rate was 61 %. Of these, 85 % (168/197) of the respondents returned both the first and second questionnaire. After exclusion of participants due to incorrect recruitment according to the age (*n* = 4) and language-criterion (*n* = 1), a large number of uncompleted questions (*n* = 2), a missing first questionnaire (*n* = 2), anonymous returned questionnaire (*n* = 1) or return outside the time interval of data inclusion (*n* = 11); data of 176 eligible patients (54 % of the invited patients) was available for analysis.

The mean age of participants was 50.5 years (range 30–60); 30.7 % were men and the vast majority were in a significant relationship (86.9 %) and had children (median: 2, range: 1–4). These and further socio-demographic characteristics are displayed in Table 1.

**Table 1:** Socio-demographic and medical characteristics participants and non-responders

	Participants (*N* = 176)				Non-responders (*n* = 122)^a^			
	M	SD	n	%	M	SD	n	%
Socio-demographic Characteristics								
Age	50.5	7.2			51.6	8.2		
Sex								
Men			54	30.7			38	31.1
Woman			122	69.3			83	68.0
Relational status								
Single			20	11.4				
Partner, married or living together			141	80.1				
Partner, not married or living together			12	6.8				
Widowed			3	1.7				
Having children			148	84.1				
Family members	11.9	10.8						
Supportive family members	6.6	4.2						
Supportive friends	13.5	12.6						
Graduation level								
Elementary school			13	7.4				
High school			101	57.7				
Graduate school			53	30.3				
University			8	4.6				
Job occupation								
Employed			41	23.3				
Work interruption/on sick leave			91	51.7				
Unemployed			12	6.8				
Disabled			20	11.4				
Housewife/houseman			6	3.4				
Retired			6	3.4				
Monthly house hold income								
< € 1500			51	30.7				
€ 1500–€ 3000			79	47.6				
> € 3000			36	21.7				
Medical characteristics								
Type of treatment								
Surgery			138	81.7			94	84.7
Radiotherapy			104	61.2			52	46.8
Chemotherapy			109	64.5			57	51.8
Hormone therapy			58	34.3			27	24.3
Immune therapy			1	0.6			1	0.9
Concomitant radio-chemotherapy			18	10.7			16	14.4
Bone marrow transplantation			0	0.0			0	0.0
Isotopes			1	0.6			0	0.0
Other treatment			5	3.0			6	5.5
Time since diagnosis (weeks)^b, c^	62.8	104.5			−	−		
Phase of care trajectory								
Active treatment phase			115	65.3				
Completion of treatment			13	7.4				
Follow-up phase			47	26.9				

*Abbreviations*: M mean, SD standard deviation, n number of participants

^a^Data of only 117 out of 128 non-responders received, ^b^Date of questionnaire completion or diagnosis missing for some participants, mean time since diagnosis based on *n* = 158, ^c^Time since diagnosis unknown for non-responders, since date of invitation to participate in the research was not registered

The sample was characterized by a wide variation in cancer diagnoses: respectively, breast (55.7 %), colorectal (11.9 %), prostate (6.3 %), head-neck (4 %), testes (2.8 %), lung (1.7 %), malign melanoma (1.7 %), brain (1.7 %), esophagus (1.7 %), liver-gall-bladder (1.1 %), cervix (1.1 %), uterus body (1.1 %), ovarian (1.1 %), kidney (1.1 %), bladder (0.6 %), thyroid (0.6 %), stomach (0.6 %) and bone cancer (0.6 %). Further medical data are shown in Table 1.

Age, type and date of diagnosis and treatment (s) of non-participants were collected anonymously to explore the representativeness of the research sample (Table 1). As compared with participants, the group of non-responders was heterogeneous with respect to cancer diagnosis: within 16 different types of diagnoses, the four most common were: breast (34.4 %), colorectal (12.3 %), malign melanoma (9.8 %) and prostate cancer (5.7 %).

### Feasibility

#### CARES item characteristics

The mean number of missing answers on the QOL-items in participants’ CARES completion was 2.3 (SD 5.0). Telephone follow-up with participants revealed that missing answers were mainly due to the accidental skipping of items or participants’ not deeming an item (s) to be applicable to them. Examples of reasons given are as follows: “I am a widow and I don’t have sex anymore, so I didn’t answer on the statement ‘I do not feel sexually attractive’”; “I don’t own a car so I couldn’t answer the question on having difficulty with driving”; “I couldn’t answer the question ‘I have difficulty preparing meals’, because my wife is the one that cooks at home, I never do”. Outliers of 66 and 58 missing answers are found on item 18 and 80. This was due to missing response categories in the first printed version of the questionnaire.

The mean number of missing answers on the Help-items of the CARES was 12.4 (SD 21.5)-considerably higher than the number of missing values on corresponding QOL-items. Participants answered the Help-questions by marking the response categories in three different ways: by marking each ‘yes’ or ‘no’ for each Help-question individually; by circling the words ‘yes’ or ‘no’ on the top of the column; or by circling the whole column of yes-or no-responses on the page. Only 49 participants (27.8 %) had no missing answers on the help-items (93–132 items). Both concurrent needs assessment measures had a lower number of missing values. For the one single help-question joining the DT and PL only four participants (2.3 %) did not complete the help-question. For the Care Needs Questionnaire only four to 10 participants (2.3–5.7 %) did not complete the life domain specific help-question.

#### Patients’ experiences in completing the CARES

On average participants needed 31 min (SD = 24.209) to complete the CARES. Ninety percent felt this to be acceptable, 10 % thought this was too long and too time consuming. Participants in this study had to complete the CARES on paper. Seventy-three percent preferred this option while 21 % would have preferred an electronic version. The reasons mentioned for preferring paper were as follows: easier for concentration; limited burden on the eyes; the ability to fill in anywhere; the lack of familiarity with the computer. On the other hand, reasons for preferring an electronic version for the computer or tablet included environmental concerns, the completion time of a screening and easier processing of results.

### Reliability

#### Internal consistency and test-retest reliability

To explore the reliability of the CARES total, sub- and summary scales, alpha coefficients were calculated (Table 2). The mean for all subscales was .79 (range .21–.94). For the five summary scales of the CARES the mean of alpha coefficients was .92 (range.87–.96).

**Table 2:** Reliability ratings and factor pattern for the flemish CARES (*N* = 176)

	Internal Consistency	Test-Retest Correlation		Factor loadings^b^				
**Global CARES**, **sub**-**and summary scales**	α	n	r^a^	Factor 1	Factor 2	Factor 3	Factor 4	Factor 5
**PHYSICAL**	.**93**	**156**	.**90**					
Ambulation	.83	158	.84	.**749**			.371	
Activities of daily living	.85	158	.83	.**795**				
Recreational Activities	.81	157	.73	.**729**				
Weight Loss	.74	157	.68	.**733**				
Difficulty Working	.93	152	.81	.**728**				
Pain	.71	156	.77	.**430**			.448	.369
Clothing	.94	156	.76	.**344**			.347	.322
**MEDICAL INTERACTION**	.**87**	**156**	.**70**					
Problems Obtaining Info from Medical Team	.85	156	.61					.**836**
Difficulty Communicating with Medical Team	.86	157	.69		.540			.**397**
Control of Medical Team	.77	157	.69					.**776**
**MARITAL**	.**90**	**133**	.**84**					
Communication with Partner	.93	155	.82		.469	.**636**		
Affection with partner	.85	155	.74			.**851**		
Interaction with Partner	.88	155	.80			.**705**		
Overprotection by Partner	.56	155	.53	.313		.**461**		
Neglect of Care by Partner	.21	155	.63			.**574**		.326
**PSYCHOSOCIAL**	.**96**	**156**	.**91**					
Body Image	.84	157	.80		.**385**		.549	
Psychological Distress	.86	157	.89	.302	.**589**		.466	
Cognitive problems	.89	157	.81	.429	.**325**		.413	
Difficulty Communicating with friends/relatives	.83	158	.77		.**610**			
Friends/Relatives Difficulty Interacting	.73	156	.65		.**538**		.324	
Anxiety in Medical Situations	.89	156	.86		.**772**			
Worry	.83	157	.84	.359	.**664**			
Interaction with Children	.78	155	.73	.330	.**525**			
At Work Concerns	.81	155	.67		.**566**			
**SEXUAL**	.**92**	**142**	.**89**					
Sex Interest	.82	156	.85			.460	.**648**	
Sexual Dysfunction	.92	154	.84				.**533**	
**CARES TOTAL**	.**88**	**158**	.**92**					

^a^all r significant at 0.01 level (2-tailed), ^b^Only factor loadings ≥ .30 are presented, factor loadings of facets belonging to each of the five CARES summary scales are in bold

The average timespan between the first and second CARES completion of participants was 12.62 days (SD 9.3). Spearman’s rho correlations between the two completions were computed to explore test-retest reliability. For all subscales high correlations were found ranging from .53 to .89 with an average of .76. Test-retest correlations for the five summary scales were all high, with an average of .85 (Table 2). The CARES total scores had a high correlation of .92. These reliability ratings demonstrate an excellent test-retest reliability of the Flemish CARES.

### Validity

#### Content validity

The majority of participants rated all life domains addressed in the CARES to be important to very important in a QOL and needs assessment tool (Table 3). Most of them (90 %) evaluated the content of the CARES to be complete. The three main areas where deficiencies were cited were the feeling of loneliness in the disease experience, financial concerns due to the disease and treatment and the lack of questions addressing the coping of patients’ loved ones.

**Table 3:** Participants’ evaluation of the content of the CARES (*N* = 159)

How important do you think several areas of well-being are to be addressed in the CARES, when the purpose is to comprehensively assess quality of life and care needs with the instrument?	Response distribution^a^			
	Very important	Important	Not so important	Totally not important
Physical well-being	90 (56.6 %)	62 (39.0 %)	2 (1.3 %)	0 (0.0 %)
Medical interaction	93 (58.5 %)	59 (37.1 %)	3 (1.9 %)	0 (0.00 %)
Relational well-being	82 (51.6 %)	59 (37.1 %)	7 (4.4 %)	1 (0.6 %)
Psychosocial well-being				
Body image	31 (38.4 %)	82 (51.6 %)	12 (7.5 %)	0 (0.00 %)
Problems with memory and/or concentration	68 (42.8 %)	79 (49.7 %)	7 (4.4 %)	0 (0.00 %)
Stress, fear, concerns on disease and treatment	84 (52.8 %)	66 (41.5 %)	4 (2.5 %)	0 (0.00 %)
Dealing with family and friends	63 (39.6 %)	79 (49.7 %)	12 (7.5 %)	0 (0.00 %)
Dealing with the children	78 (49.1 %)	66 (41.5 %)	7 (4.4 %)	0 (0.00 %)
Concerns about work	53 (33.3 %)	77 (48.4 %)	19 (11.9 %)	3 (1.9 %)
Sexual interest and functioning	43 (27.0 %)	79 (49.7 %)	27 (17.0 %)	2 (1.3 %)
Miscellaneous				
Financial difficulties	51 (32.1 %)	80 (50.3 %)	18 (11.3 %)	5 (3.1 %)
Finding a partner	22 (13.8 %)	52 (32.7 %)	37 (23.3 %)	27 (17.0 %)
Difficulties with regard to treatment	67 (42.1 %)	66 (41.5 %)	12 (7.5 %)	4 (2.5 %)
Was there a topic missing in the CARES that you find important in an assessment on psychosocial concerns and care needs?			No	Yes
			132 (89.80 %)	15 (10.20 %)

^a^Percentages do not count up to 100 % due to missing values.

#### Concurrent validity

Spearman rho correlations for CARES total, summary scores and convergent measures were in the expected directions (Additional file 1). The KPS and CARES physical scale have a large negative correlation (*r* = -.67). HADS scores and the CARES psychosocial scale are strongly positive related (*r* = .75 and *r* = .64). From the SSL only the D-subscale had a significant moderate correlation with the Psychosocial CARES summary scale (*r* = .43). The Marital and Sexual CARES summary scales are moderate to strongly positive related to the MMQ-M (*r* = .48) respectively MMQ-S (*r* = .55). Also the large correlations of the CARES Total score with the EORTC-QLQ-C30 (*r* =−.56 and *r* =−.53) and DT (*r* = .63) confirm the concurrent validity of the CARES.

#### Construct validity

There are *intercorrelations* of .32–.60 between CARES summary scales, indicating that these measure related but different dimensions of concerns and care needs. The summary scales all have a high correlation with the CARES Total, indicating an important role in the quality of life disruption measured by the CARES (Additional file 1).

To ensure that the data were suitable for *factor analysis* standard diagnostic tests were run each time. Both the Kaiser-Meyer-Olkin (KMO) test of sampling adequacy criterion (KMO ≥ .6) and Bartlett’s test of sphericity criterion (*p* < .05) were fulfilled and indicated factorability of the data.

Firstly, the CARES subscales were explored. For the items of the physical summary scale six factors were found. Medical interaction-items loaded on three factors, psychosocial-items on nine, marital-items on four and the items of the sexual summary scale on two factors (Additional file 2).

Secondly, the summary scales were explored. Based on Kaiser’s criterion (eigenvalue ≥1) seven factors were distinguished with the PCA, explaining a total of 65.5 % of the variance. However, based on Catell’s scree test, only the first five factors should be retained to get a good fitted model of factors explaining the variance in our data set. Subsequently a PCA with varimax rotation and fixed number of five factors was conducted resulting in the factor solution visualized in Table 2. The resulting factor solution approximately corresponds to the subdivision of the CARES in the five summary scales: physical, interaction with the medical team, marital, psychosocial and sexual.

## Discussion

This study explored the validity of the Flemish CARES version, resulting in a positive evaluation of the instrument.

The small number of missing answers on CARES’ QOL-items indicates that the items were clear to the vast majority of participants, which supports the feasibility of the instrument for wider application or use among Flemish cancer patients. Participants also reported positive experiences with the content and completion time of the CARES. The number of missing answers on the Help-items of the CARES is relatively higher. The question is raised whether if it is relevant to have a help-question for each QOL-item. Possibly circling requires a great effort of participants, resulting in a larger number of missing answers, while domain specific help-questions could be sufficient to reveal patients supportive care needs. The smaller number of missing answers on the concurrent needs assessment instruments, may indicate that a simplified help-questioning could be more feasible. For example the 93–132 help-items could be reduced to several life domain specific help-questions presented each time after a group of QOL-items. Although this aspect could use some improvement, the majority of the participants are in favor of the use of a QOL and needs assessment tool like the CARES in clinical practice.

The CARES provides a total score and five domain specific scores, which all demonstrated high reliability. The two subscales with low alpha coefficients ‘Overprotection by Partner’ (α = .56) and ‘Neglect of care by partner’ (α = .21) are scales with only two items. Having fewer items in a scale is known to have a lowering effect on the alpha coefficient. These reliability ratings correspond to those of the original CARES.

The results of the PCA confirm the existence of five distinguishable components of QOL measured with the Flemish CARES, similar to the physical, medical interaction, relational, psychosocial and sexual summary scale of the original instrument. However, some subscales have double loadings. PCA should be reproduced as soon as a larger research sample is available.

Concurrent validity of the CARES and its’ summary scales with several instruments was confirmed with moderate to high correlations. This implies that the CARES could be used to obtain a comprehensive summary of patients’ overall QOL and care needs from their own perspective instead of having to combine several other patient reported outcome tools.

Limitations of this study should be noted. Rules-of-thumb for the number of subjects included in factor analysis vary from four to 10 subjects per item of the questionnaire [32]. With 176 participants our research sample is rather limited. However, the factor pattern of the original instrument was already known and even with our relatively small number of participants the original factor solution could be replicated. The CARES was developed for cancer patients in general, though the representativeness of our sample could be questioned. To pursue representativeness, recruitment was performed in several departments of the participating hospitals. This resulted in a heterogeneous sample of 25–60 years aged cancer patients, with breast, colorectal, prostate and head-neck cancer as most common cancer types. This matches the national statistics [54], and characteristics of our group of non-responders. Non-responders seem to have undergone less invasive treatment (Table 1). However, there is a lack of further information, for example on ‘time since diagnosis’, to make a detailed comparison. The selection of patients aged between 25–60 years to capture the adult population of cancer patients, was an inherent limitation as it limited the generalizability of results since approximately three-quarters of cancers are diagnosed in people aged over 60 years. The utility and validity of the Flemish CARES should further be explored in patients aged older than 60 years, before the instrument is implemented in clinical practice.

While this study demonstrates rigor of the Flemish CARES version across key psychometric properties, we must acknowledge that other indices were not explored, e.g. known groups comparison, predictive validity, responsiveness. Consequently, future studies that focus on these aspects could strengthen the evidence of the validity of the Flemish CARES version.

## Conclusions

This study confirms the Flemish CARES version to be a comprehensive and feasible QOL and needs assessment instrument with good psychometric properties. Consequently, the Flemish CARES can be used in further research to assess QOL and care needs. Further translational research studies are needed to explore how the use of such a tool can be implemented efficiently in clinical practice to contribute to quality patient-centered care.

## Additional files

Additional file 1: CARES Validity Ratings. (DOC 45 kb) Additional file 2: Factor solutions exploring CARES subscales. (DOC 167 kb)

## Abbreviations

CARES: Cancer rehabilitation evaluation system
CEBAM: Belgian center for evidence-based medicine
CIPS: Cancer inventory of problem situations
DAS: Dyadic adjustment scale
DT: Distress thermometer
EORTC-QLQ-C30: European organisation of research and treatment for cancer quality of life questionnaire core 30
HADS: Hospital anxiety and depression scale
KMO: Kaiser-meyer-olkin
KPS: Karnofsky performance status scale
M: Mean
MMQ-M: Maudsley marital questionnaire
PCA: Principal component analysis
QOL: Quality of life
SD: Standard deviation
