# Supplementary material for: Validation of the flemish CARES, a quality of life and needs assessment tool for cancer care
Source: BMC Cancer. 2016 Aug 30;16(1):696. doi: 10.1186/s12885-016-2728-9 (PMC5006609; doi:10.1186/s12885-016-2728-9)
Supplement: Additional file 1: — CARES Validity Ratings. (DOC 45 kb) [file 12885_2016_2728_MOESM1_ESM.doc]

**Concurrent validity**

**TABLE A1.** Correlations of CARES Total and Summary scores with Concurrent Validity Measures

| Concurrent Validity Measures | CARES  Total | Physical | Medical  Interaction | Psychosocial | Marital | Sexual |
| --- | --- | --- | --- | --- | --- | --- |
| KPS | **-.50**** | **-.67**** | -.15* | -.38 | -.23** | -.39** |
| HADS-A | **.68**** | .48** | .36** | **.75*** | .48* | .38** |
| HADS-D | **.67**** | .60** | .32** | **.64*** | .45* | .45** |
| SSL-I | **.09** | .17* | -.19* | **.07** | -.02 | -.001 |
| SSL-D | **.38**** | .18* | .33** | **.43*** | .37** | .25** |
| MMQ-M | **.25**** | .11 | .18* | .18* | **.48**** | .26** |
| MMQ-S | **.54**** | .36** | .27** | .39** | .54** | **.55*** |
| EORTC-QOL-C30 General Health | **-.56**** | -.71** | -.19* | -.36** | -.26** | -.38** |
| EORTC-QPM-C30 Quality of Life | **-.53**** | -.67** | -.15* | -.37** | -.21** | -.28** |
| DT | **.63**** | .64** | .21** | .54** | .37** | .43** |
| *Abbreviations*: CARES (Cancer Rehabilitation Evaluation System), KPS (Karnofsky Performance status Scale), HADS-A and -D (Hospital Anxiety and Depression Scale, Anxiety and Depression), SSL-I and -D (Social Support List, Interactions and Discrepancies), MMQ-M and -S (Maudsley Marital Questionnaire,Marital and Sexual), EORTC-QOL-C30 (European Organisation of Research and Treatment for Cancer Quality of Life Questionnaire Core 30), DT (Distress Thermometer).  Correlations of interest are in bold.  ** r significant at 0.01 level (2-tailed)  * r significant at 0.05 level (2-tailed) | | | | | | |

**Construct validity**

**TABLE A2.** Intercorrelations of CARES Total and Summary Scales (N=176)

|  | CARES  Total | Physical | Medical | Marital | Psychosocial |
| --- | --- | --- | --- | --- | --- |
| Physical | .80 |  |  |  |  |
| Medical | .49 | .32 |  |  |  |
| Marital | .71 | .42 | .42 |  |  |
| Psychosocial | .88 | .56 | .49 | .60 |  |
| Sexual | .71 | .48 | .29 | .51 | .58 |
| * all r significant at 0.01 level (2-tailed) | | | | | |
