# Supplementary material for: Validation of the flemish CARES, a quality of life and needs assessment tool for cancer care
Source: BMC Cancer. 2016 Aug 30;16(1):696. doi: 10.1186/s12885-016-2728-9 (PMC5006609; doi:10.1186/s12885-016-2728-9)
Supplement: Additional file 2: — Factor solutions exploring CARES subscales. (DOC 167 kb) [file 12885_2016_2728_MOESM2_ESM.doc]

| **TABLE A3.** Varimax Rotation Factor Pattern of the Physical summary scale items (N=176) | | | | | | |
| --- | --- | --- | --- | --- | --- | --- |
| **CARES items^b^** | **Factor loadings^a^** | | | | | |
|  | 1 | 2 | 3 | 4 | 5 | 6 |
| 1.Diff. bend or lift | **.650** |  |  |  | .341 |  |
| 2.Diff. walk/move around | **.641** | .355 |  |  | .324 |  |
| 3.Diff. do physical activ. | **.687** |  |  |  |  |  |
| 4.Reduction in energy | **.530** |  | .323 |  |  |  |
| 5.Diff. driving | **.710** |  |  | .340 |  |  |
| 6.Diff. household chores | **.715** | .331 |  |  |  |  |
| 7.Diff. bathe. brush. groom | **.712** |  |  |  |  | .310 |
| 8.Diff. prepare meals | **.630** | .377 |  | .353 |  |  |
| 9.No interest recreat. active. |  | **.729** |  |  |  |  |
| 10.Not engage recreat. active. | .429 | **.640** |  |  |  |  |
| 11.Not enough enjoyable activ. |  | **.664** |  |  |  |  |
| 12.Diff. planning active. |  | **.779** |  |  |  |  |
| 13.Cannot gain weight |  |  |  |  |  | **.713** |
| 14.Continue to lose weight |  |  |  | **.412** |  | **.743** |
| 15.Food unappealing |  |  |  | **.803** |  |  |
| 16.Food tastes bad |  |  |  | **.763** |  |  |
| 17.Diff. swallowing |  |  |  | **.686** |  |  |
| 19.Cancer interferes work | .377 | **.480** |  |  |  |  |
| 20.Frequently has pain | .380 |  |  |  | **.725** |  |
| 21.Chronic pain scars/surgery |  |  |  |  | **.676** |  |
| 22.Pain not controlled medication |  |  |  |  | **.780** |  |
| 23.Pain controlled medication |  |  |  |  | **.502** |  |
| 24.Clothes not look good |  |  | **.912** |  |  |  |
| 25.Clothes not fit |  |  | **.898** |  |  |  |
| 26.Diff. find clothes |  |  | **.882** |  |  |  |
| ^a^ Only factor loadings ≥ .30 are presented. factor loadings of facets belonging to each of the CARES subscales are in bold. ^b^ Order of items is determined by the original order of the subscales in the CARES. | | | | | | |

| **TABLE A4.** Varimax Rotation Factor Pattern of the Sexual summary scale items (N=175) | | |
| --- | --- | --- |
| **CARES items^b^** | **Factor loadings^a^** | |
|  | 1 | 2 |
| 74.Doesn’t feel sex. attractive | .510 | **.670** |
| 75.Thinks not sexually attractive to partner(s) |  | **.927** |
| 76.Not interested in having sex | .798 | **.331** |
| 77.Doesn’t think partner(s) interested in sex |  | **.828** |
| 99.Frequency of seks decreased | **.811** | .342 |
| 100.Diff. become sexually aroused | **.913** |  |
| 101.Diff. with erection (males) / Diff. lubrication (females) | **.847** |  |
| 102.Diff. reach orgasm | **.883** |  |
| ^a^ Only factor loadings ≥ .30 are presented. factor loadings of facets belonging to each of the CARES subscales are in bold. ^b^ Order of items is determined by the original order of the subscales in the CARES. | | |

| **TABLE A5.** Varimax Rotation Factor Pattern of the Medical Interaction summary scale items (N=176) | | | |
| --- | --- | --- | --- |
| **CARES items^b^** | **Factor loadings^a^** | | |
|  | 1 | 2 | 3 |
| 27.Medical team withholds info |  | **.877** |  |
| 28.Doctors don’t explain what do |  | **.843** |  |
| 29.Nurses don’t explain what do |  | **.860** |  |
| 30.Diff. ask doctors questions | **.864** |  | .303 |
| 31.Diff. ask nurses questions | **.799** |  | .304 |
| 32.Diff. express feelings doctor/nurses | **.834** |  |  |
| 33.Diff. tell doctor new symptoms | **.764** |  |  |
| 34.Diff. understand doctor about cancer |  |  | **.775** |
| 35.Diff. understand nurses about cancer |  |  | **.852** |
| 36.Wants more control over doctor |  | .618 | **.543** |
| 37.Wants more control over nurses |  |  | **.758** |
| ^a^ Only factor loadings ≥ .30 are presented. factor loadings of facets belonging to each of the CARES subscales are in bold. ^b^ Order of items is determined by the original order of the subscales in the CARES. | | | |

| **TABLE A6.** Varimax Rotation Factor Pattern of the Marital summary scale items (N=153) | | | | |
| --- | --- | --- | --- | --- |
| **CARES items^b^** | **Factor loadings^a^** | | | |
|  | 1 | 2 | 3 | 4 |
| 103.Diff. talk feelings | **.786** |  | .372 |  |
| 104.Diff. talk fears | **.825** |  |  |  |
| 105.Diff. talk happen after death | **.804** |  |  | .344 |
| 106.Diff. talk future | **.834** |  |  |  |
| 107.Diff. talk cancer | **.888** |  |  |  |
| 108.Diff. talk wills/financial matters | **.823** |  |  |  |
| 109.Doesn’t feel like embrace. etc. |  |  | **.739** |  |
| 110.Partner no feel like embrace. etc. |  |  | **.830** |  |
| 111.No interest in touch partner |  |  | **.714** |  |
| 112.Partner no interest in touch |  |  | **.774** |  |
| 113.Not get along as well usual |  | **.825** |  |  |
| 114.Upset with other more often |  | **.869** |  |  |
| 115.So much time together. on nerves |  | **.632** |  |  |
| 116.More distant then usual |  | **.778** | .302 |  |
| 117.Partner not let do activ. capable of |  |  |  | **.771** |
| 118.Partner provides too much care |  |  |  | **.751** |
| 119.Partner takes too little care |  | .575 |  |  |
| 120.Diff. ask partner to take care | .350 | . |  | .493 |
| ^a^ Only factor loadings ≥ .30 are presented. factor loadings of facets belonging to each of the CARES subscales are in bold. ^b^ Order of items is determined by the original order of the subscales in the CARES. | | | | |

| **TABLE A7.** Varimax Rotation Factor Pattern of the Psychosocial summary scale items (N=176) | | | | | | | | | |
| --- | --- | --- | --- | --- | --- | --- | --- | --- | --- |
| **CARES items^b^** | **Factor loadings^a^** | | | | | | | | |
|  | 1 | 2 | 3 | 4 | 5 | 6 | 7 | 8 | 9 |
| 38.Embarrassed to show body |  |  |  |  | **.847** |  |  |  |  |
| 39.Uncomfor. show scars |  |  |  |  | **.830** |  |  |  |  |
| 40.Uncomfor. with body changes |  |  |  |  | **.661** |  |  |  |  |
| 41.Frequently anxious | .414 | **.559** |  |  |  |  |  |  |  |
| 42.Frequently depressed |  | **.710** |  |  |  |  |  |  |  |
| 43.Frequently angry |  | **.752** |  |  |  |  |  |  |  |
| 44.Frequently upset |  | **.738** |  |  |  |  |  |  |  |
| 45.Frequently overwhelmed by cancer | .399 | **.524** |  |  |  | .321 |  |  |  |
| 46.Diff. sleep |  |  |  | .455 |  | .520 |  |  |  |
| 47.Diff. concentrating |  |  |  | **.828** |  |  |  |  |  |
| 48.Diff. remembering |  |  |  | **.813** |  |  |  |  |  |
| 49.Diff. thinking clearly |  |  |  | **.816** |  |  |  |  |  |
| 50.Diff. tell frnd/rel. to come less often |  |  | **.747** |  |  |  |  |  |  |
| 51.Diff. tell frnd/rel. to leave when not well |  |  | **.742** |  |  |  |  |  |  |
| 52.Diff. ask frnd/rel. to do fun things |  |  | **.731** |  |  |  |  |  |  |
| 53.Don’t know what to say to frnd/rel. |  |  |  |  |  |  |  | .750 |  |
| 54.Diff. ask frnd/rel. help |  |  | **.659** |  |  |  |  |  |  |
| 55.Diff. tell frnd/rel. about cancer | .592 |  |  |  | .372 |  |  | .449 |  |
| 56.Diff. ask frnd/rel. to come more |  |  | **.727** |  |  |  |  |  |  |
| 57.Frnd/rel. say look well when not | .356 |  | .435 |  |  |  |  |  | .313 |
| 58.Frnd/rel. withhold information |  |  |  |  |  |  |  | .721 |  |
| 59.Frnd/rel. avoid talk cancer |  |  |  |  |  |  |  |  | .699 |
| 60.Frnd/rel. do not visit enough |  |  |  |  |  |  | **.889** |  |  |
| 61.Frnd/rel. do not call enough |  |  |  |  |  |  | **.836** |  |  |
| 62.Frn/rel. uncomfor. visiting |  |  |  |  |  |  | **.325** |  | .558 |
| 63.Frnd/rel. diff. talk about cancer |  |  |  |  |  |  | **.527** | **.**303 | .566 |
| 64.Uncomfor. see patients get treat. | **.765** |  |  |  |  |  |  |  |  |
| 65.Nervous going to hospital | **.755** |  |  |  |  |  |  |  |  |
| 66.Nervous wait to see doctor | **.765** |  |  |  |  |  |  |  |  |
| 67.Nervous wait for test results | **.717** |  |  |  |  | .314 |  |  |  |
| 68.Nervous have diagnostic tests | **.462** | .493 |  |  |  |  |  |  |  |
| 69.Nervous get blood drawn | **.680** |  |  |  |  |  |  |  |  |
| 70.Worry whether treatments work | .453 | .359 |  |  |  | **.594** |  |  |  |
| 71.Worry whether cancer progress | .355 | .386 |  |  |  | **.610** |  |  |  |
| 72.Worry not able to care for self |  |  |  |  |  | **.696** |  |  |  |
| 73.Worry how family will manage |  |  |  |  |  | **.660** |  |  |  |
| ^a^ Only factor loadings ≥ .30 are presented. factor loadings of facets belonging to each of the CARES subscales are in bold. ^b^ Order of items is determined by the original order of the subscales in the CARES. | | | | | | | | | |
